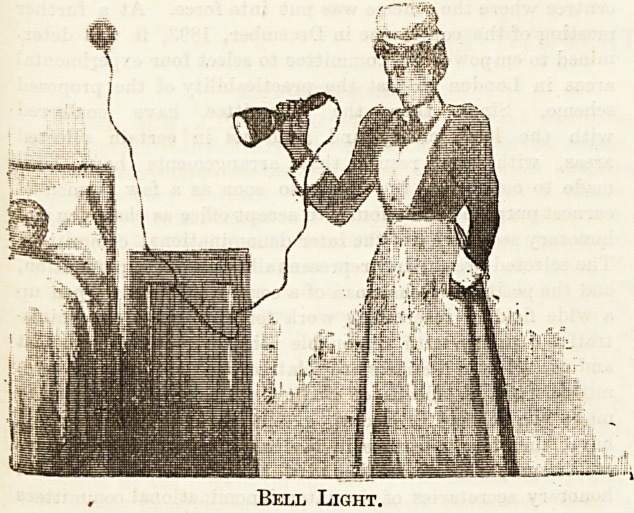# Practical Departments

**Published:** 1894-11-10

**Authors:** 


### PRACTICAL DEPARTMENTS.

### ELECTRIC LIGHTING FOR HOSPITALS.—(*Continued.*)

The wards at Middlesex Hospital are, as we have said, plentifully supplied with lights. In addition to those down the centre of every ward, there are bracket lamps beside each bed, such as the one shown in the first illustration, giving separate illumination to each patient; and a delightful arrangement is that of the bell lamp for hand use, also provided for each bed, and one which, while concentrating a brilliant light where needed, is shaded so as to avoid any unnecessary flashing in the patient's eyes. Thus all carrying to and fro of lights is avoided, a comfort only to be fully appreciated by those who have had practical experience of the trials and dangers which attach to the use of the ordinary oil lamp.

We spoke last week of the comparative cost of gas and electric light, and mentioned that at Middlesex the current is obtained from the Metropolitan Electric Light Company. It is possible that at some future time the hospital may arrange to have its own plant, and thus effect a certain saving. The difficulty to be overcome in this case is how to place the engines where the noise and vibration will not be a source of discomfort to the sick occupants of the wards. This will be more easily done, probably, at Middlesex than at many hospitals, where the premises are smaller, and there is no possibility of extension. In considering the whole question of expense, it must not be overlooked that the figures we have quoted in regard to the Middlesex Hospital date already two years back, and that with every coming 'year the installation of electric light may be expected to cost less.

Bracket Lamp.

For the regulation of the general expenditure upon lighting, Mr. Melhardo has organised a system of supervision by which the weekly consumption in each several department is entered in a book kept for the purpose, a reference to which shows immediately any unusually high figures. Inquiries can then be made of the official responsible, and all waste promptly checked.

Bell Light.

On the whole we think it may safely be said that the experiment carried out at Middlesex Hospital has amply justified in the result any increased expenditure.

Cooking by electricity is an achievement still in its infancy, but if sanguine people are right in the future they prophesy for it, we may perhaps expect to see its application to institutions with satisfactory economical results.

(*To be continued.*)

## Figures and Tables

**Figure f1:**
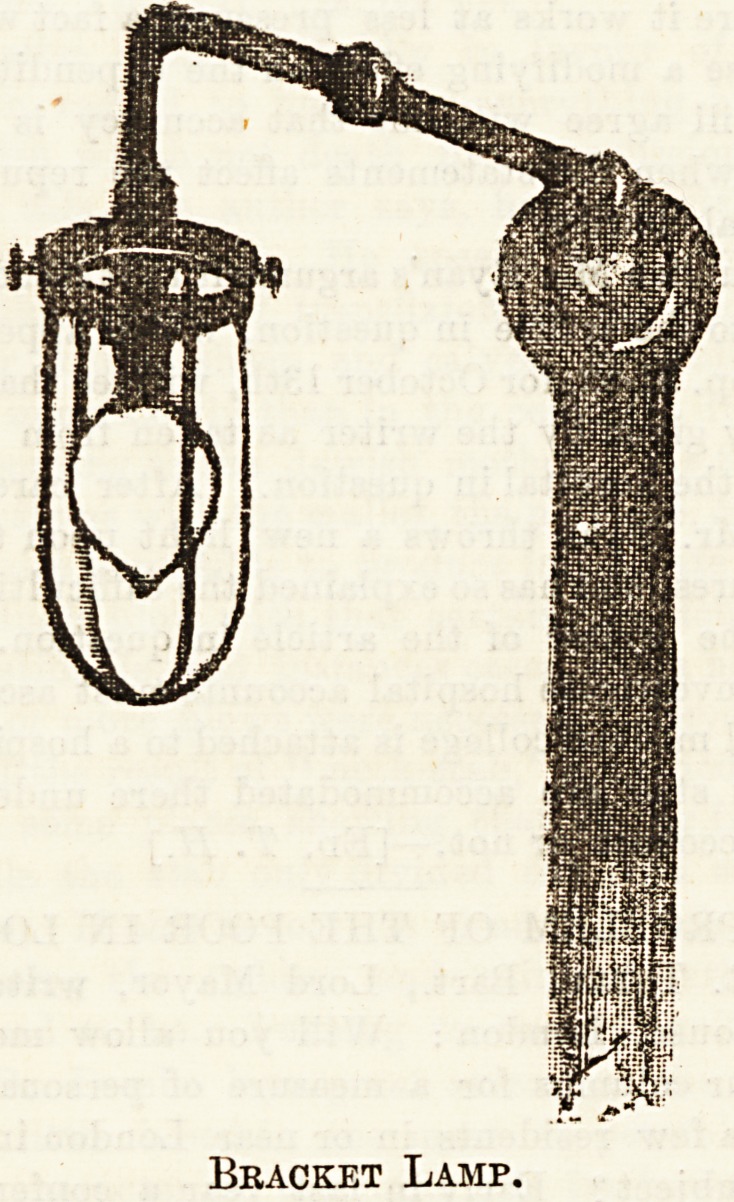


**Figure f2:**